# A case report of osteopenia of prematurity

**DOI:** 10.1016/j.radcr.2024.09.030

**Published:** 2024-09-25

**Authors:** Vahideh Hosseinzadeh, Elias mazrooei rad, Aida Alirezaee

**Affiliations:** a Pediatric resident, Mashhad University of Medical Sciences, Mashhad, Iran; b Biomedical Engineering Department, khavaran institute of higher education, Mashhad, Iran; c Student Research Committee, Mashhad University of Medical Sciences, Mashhad, Iran

**Keywords:** Osteopenia, Prematurity, Alkaline phosphatase, Bone metabolic disease

## Abstract

The girl neonate with 1500 g, was transferred to the neonatal intensive care unit due to tachypnea and prematurity. She received supportive and therapeutic care in the course of hospitalization. Due to the high level of alkaline phosphatase in the examinations and x-rays of the wrist, premature osteopenia was diagnosed and she was treated with high doses of calcium and phosphorus. Alkaline phosphatase was measured weekly in the course of treatment, with its downward trend indicating an appropriate response to treatment. Although osteoporosis is a common and recurrent disease in premature neonate, but it can be decrease with preventing factors that lead to premature infants and by providing necessary screening and proper timely treatment with nutritional supplements and prevented the progress of the disease.

## Background

Osteoporosis is a common disease in premature neonates due to inadequate calcium and phosphorus intake after birth. Calcium and phosphorus in breast milk are insufficient for normal bone mineralization in neonates with low birth weight. Osteopenia and its symptoms may be seen incidentally on radiographs [1].This disease can cause long bones and rib fractures. In most cases, 80% of mineral accumulation in the fetus occurs in the third trimester. In comparison, the increase in the concentration of minerals in the fetus helps in its rapid increase in the growth of bones and compensates for the decrease in calcium [2]. The mother is the major source of minerals in fetal life. Parathyroid hormone and parathyroid hormone peptide play an important role in mineral physiology during fetal life. At birth, the transport of minerals through the placenta is interrupted by cutting the umbilical cord. After birth, the growth of the kidneys and intestines leads to an increase in calcium levels. Premature babies suffer from osteoporosis due to significant delay in intestinal maturation and increased demand for minerals. Most of the formation of bone mineralization occurs in late pregnancy. Accumulation of minerals is stopped through premature birth [3]. Postnatal mineral homeostasis requires balancing the function of parathyroid hormones, calcitonin and vitamin D in the target organs. Preterm birth, asphyxia, acidosis and long-term total parenteral nutrition (TPN) increase the risk of imbalance and bone metabolic disease in these infants. Bone regrowth and regeneration is very complex, which is influenced by many factors, and bone health depends on an adequate balance between all these factors [4]. Bone health depends on an adequate balance between mechanical nutritional and hormonal factors that are more effective in the mineralization process. Osteoporosis usually has a genetic or idiopathic origin (primary osteoporosis). Other risk factors for osteoporosis (secondary osteoporosis) may include maternal or pregnancy factors, endocrine changes, the use of some antagonists of bone metabolism drug and chronic diseases (kidney or liver failure malabsorption, collagen or metabolic diseases or nutritional deficiencies (calcium, phosphorus, vitamin D are other important risk factors. Premature neonatal Metabolic Bone Disease (MBD) is a multifactorial disorder that is seen in low birth weight infants and is very common in extreme low birth weight infants. MBD is associated with biochemical and radiological findings related to bone destruction. Various factors are involved in the development of the disease, but the underlying mechanism is calcium and phosphate depletion in preterm neonate. The diagnosis is made with laboratory and radiological findings. The best method preventing the disease is to prevent preterm birth and screening mothers at risk. It is possible to investigate the effect of prematurity on fracture up to the age of 5 years by controlling drugs [5]. In this study, we reported a case of a neonate with 29 weeks gestation who was diagnosed with prematurity osteoporosis.

## Case presentation

**Fig. 1:**
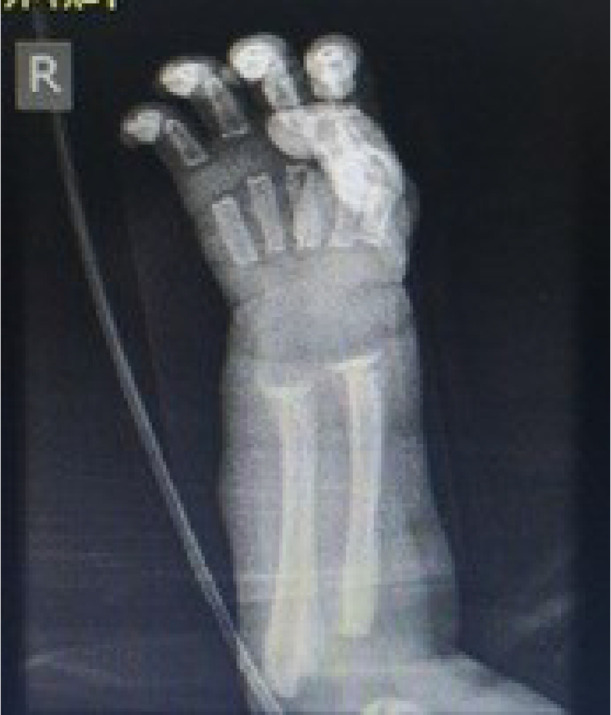
Wrist x-ray of neonatal for diagnosis prematurity osteoporosis.

**Fig. 2:**
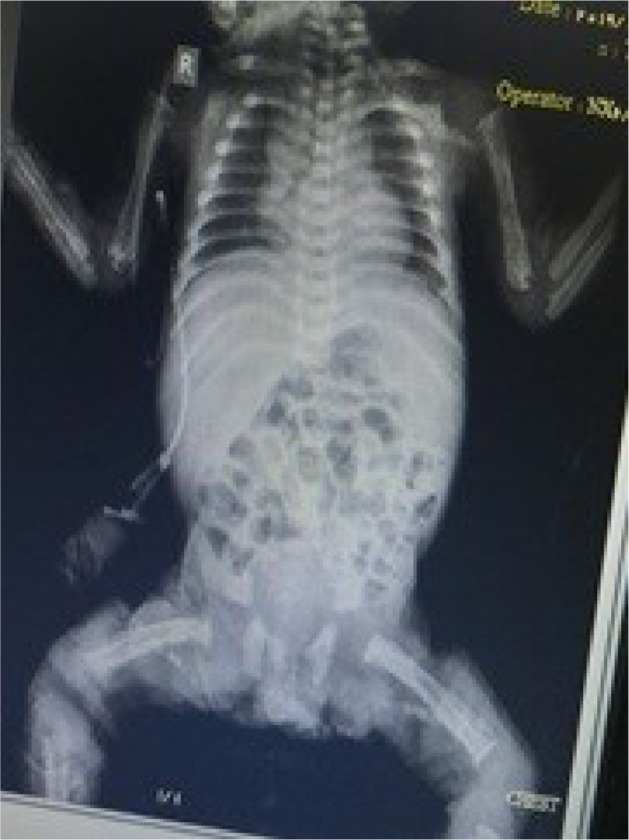
AP chest and limb X-ray.

A female neonate with birth weight 1500 g, head circumference 27 cm, height 40 cm and gestational age of 29 weeks that born due to the onset of uterine contractions. Attention to tachypnea and respiratory distress was admitted to the neonatal intensive care unit of Imam Reza Hospital in Mashhad. The neonate had a respiratory rate of 65 per minute, blood pressure of 80/60 bpm, heart rate of 160 beats per minute and temperature was 37 degrees Celsius axillary on examination, she had supra sternal and inter vertebral retraction and a 2/6 systolic murmur in the left sternal border. In the family history, father and mother had family relationship. She had no family history of certain diseases. Immediately after admission the infant was a given respiratory support due to respiratory distress and treatment with pulmonary surfactant was initiated for the neonate, as well as other therapeutic and supportive measures for premature infants. During hospitalization the patient improved respiratory status but was still treated with oxygen. She was consulted with a pediatric cardiologist and echocardiography due to suspected murmurs. In echocardiography small ASD was reported. Also, Routine tests as well as tests for premature babies were requested (Table 1). According to alkaline phosphatase 10657 mole, wrist x-ray were obtained to diagnosis of prematurity osteopenia (Fig. 1), which was evident in the evidence of rickets of prematurity. Also, to confirm the diagnosis done re-measurement of alkaline phosphatase that reported 15857 mole. In consultation with a pediatric endocrinologist, high-dose vitamin D, calcium and phosphorus treatment and supportive measures to care for bone fractures were administered. Alkaline phosphatase levels were measured weekly to respond to treatment (Table 2), which was measured Chest and limb x-rays were also taken to evaluate rib and bone fractures and to assess lung status (Fig. 2).

**Table 1:** Lab test of this neonate.

CRP	3
ESR	9
Blood culture	negative
Alp	10657
WBC	11100
lym	35 %
nut	65%
HB	12
mcv	104
Plt	156
UREA	57
Creatinin	0.7

**Table 2:** Weekly alkaline phosphatase levels.

Time	2 weeks	2 weeks	3 weeks	4 weeks	5 weeks	6 weeks
**ALP**	**10657**	**15857**	14008	**14727**	**13544**	**12000**

## Discussion and conclusions

In a study by Maleki et al. On 42 infants with Weight less than 1800 in 2009 showed that increased calcium and phosphorus intake in preterm infants could be associated with a decrease in serum alkaline phosphatase levels, which is an indicator for the diagnosis of osteoporosis. However, based on radiological criteria has no effect. Osteopenia and its symptoms may be seen incidentally on radiographs [1]. In another study carried out by Mohammadzadeh et al., in 2004, on neonates with weighing less than 2000 grams showed the effect of increasing calcium and phosphorus intake on prevention and reduction of prematurity osteopenia reported, Increasing oral intake of calcium and phosphorus in very low birth weight infants fed with breast milk can be effective in preventing premature osteopenia. In addition, supplementation of these 2 elements improves the height and weight growth of these infants in comparison, the increase in the concentration of minerals in the fetus helps in its rapid increase in the growth of bones and compensates for the decrease in calcium [2]. In a study done by Maemori et al. In 2002, on 43 neonates with weighing less than 2000 g showed increased oral intake of calcium and phosphorus in very low birth weight infants may be effective in preventing premature osteopenia. Most of the formation of bone mineralization occurs in late pregnancy. Accumulation of minerals is stopped through premature birth [3]. In addition, supplementation of these 2 elements improves the height and weight growth of these infants. Statistical analysis of the findings showed no significant difference in the incidence of osteopenia with the 2 diagnostic criteria of radiography and biochemistry. A study conducted by Faienza MF showed that optimizing complete intravenous nutrition and achieving premature oral nutrition are important goals for the prevention and management of premature neonatal metabolic diseases. Vitamin D prevents bone fractures in growing age and improves the effect of chemical phosphate as a supplement [6]. In a study by Wagner K et al. In 2019, which examined infant fractures, reported that prematurity did not increase the risk of childhood fractures and found that the use of proton pump inhibitors (PPI) drug and neonatal cholestasis increased Bone fractures occur at 5 years old. A study by Chinoy a et al., 2019, which investigated the causes of metabolic bone disease in preterm infants, showed that drugs such as steroids and diuretics lead to bone breakdown and reported progress in care and treatment Methods of feeding premature infants decline prematurity metabolic bone disease. Cholestasis is an important risk factor for developing MBD [7]. In a 2018 study by Chen W et al., on 238 neonates less than 34 weeks of gestation showed that steroid use in the mother, aminophylline and ventilator use in neonate and liver dysfunction can be effective in the development of metabolic bone disease in premature infants. A 2018 study by Ali E et al. showed that the cumulative dose of caffeine and its duration of use in preterm infants that used to prevention of prematurity apnea were as effective as steroids in the development of osteopenia of prematurity. Metabolic bone disease is a condition characterized by a decrease in bone mineral content (osteopenia) [8].

A study by Chin L in 2018 on 171 preterm infants younger than 32 weeks gestational age showed that vitamin D and phosphate supplements could be effective in improving the metabolic bone diseases of preterm infants, although further research is needed [4]. In a study by ukarapong S et al., 2017, which examined the risk factors for metabolic bone disease in preterm infants showed, cholestasis was one of the most important risk factors for the development and progression of premature bone disease. Considered the need for further studies in this field. A prospective study conducted in 2016 by Abdallah EA and colleagues on 120 preterm infants weighing less than 1500 grams evaluated serum alkaline phosphatase levels as a primary marker for osteopenia in preterm infants. the results showed Serum alkaline phosphatase is a reliable marker for predicting bone mineralization status. However, the need for radiological evaluation, especially in infants of less than 1000 g and gestational age of less than 32 weeks, was considered necessary. A study by Stalnaker KA et al. in 2016 found that combination mild physical activity and physical massage on premature infants with appropriate nutritional supplements may be effective in improving prematurity osteopenia. Since osteoporosis occurs frequently and is very common in premature neonates, it can play an important role in preventing premature neonates by preventing the causes that lead to premature birth. Also can be avoided by preventing unnecessary use of steroids and diuretics that increase the risk of prematurity osteopenia. According to some studies long-term intravenous nutrition and ventilator use are also risk factors, Therefore, it is necessary to start oral nutrition as soon as possible and Make arrangements for exhumation of preterm infant as soon as possible If the patient's condition allows. Due to the results of various studies and the role of timely screening and proper nutritional supplements, policies should be adopted for proper diagnosis, treatment and care of these neonates in neonatal intensive care units to prevent complications and progression of the disease.

## Ethical approval

We confirm that there is no conflict of interest related to this article. Ethical approval and informed consent, all procedures performed in studies involving human participants were in accordance with 1964Helsinki declaration and its later amendments or comparable ethical standards.

## Consent to participate

We agree to participate in the research project and the following is explained to us: Research may be of direct benefit to Our participation is completely voluntary. Our right to cancel the study at any time without any consequences for us.

## Consent to publish

We give my consent for the publication of identifiable details, which can include photograph(s) and/or videos and/or case history and/or details within the text (“Material”) to be published in the above Article. Therefore, anyone can read material published in the article.

## Authors' contributions

All authors contributed to the study conception and design. Material preparation, data collection and analysis were performed by Vahideh Hosseinzadeh, Elias mazrooei rad, Aida Alirezaee. The first draft of the manuscript was written by elias mazrooei rad and all authors commented on previous versions of the manuscript. All authors read and approved the final manuscript.

## Patient consent

Complete written informed consent was obtained from the patient for the publication of this study and accompanying images.
